# Vagitus Uterinus

**Published:** 1850-03

**Authors:** 


*Vagitus Uterinus.*—Dr. Landsberg, in a long article on the hydrostatic test, in Henke's 'Zeitschrift,' relates a case of this which occurred in the person of a stout muscular woman, who continued in strong labor-pains from the Saturday to the Thursday, when the liquor amnii was discharged, and a normal occipital presentation felt. While the author, finding no cause for interference, was counselling patience to a very unruly subject, he and others in the small chamber distinctly heard the voice of the child proceeding from the mother's belly, sounding just as it would if issuing from underneath a covering. It ceased after a-while; and as no progress in the labor seemed to be taking place, Dr. Landsberg delivered the woman with the forceps, of a still-born child, which, however, was soon reanimated. He observes, there was not here the large pelvis which some authors represent as always found in such cases.

He adds, that there thus may occur cases, in which, although it is evident the child has breathed, it becomes difficult or impossible to declare whether respiration commenced *in utero* and became arrested during the progress of birth, or whether it commenced first after birth, and was then obstructed by forcible prevention of the entrance of air into the passages. In most cases, we have evidence of external violence, while *vagitus uterinus* must always be considered a most rare occurrence, happening for the most part, only in very tedious labors, while clandestine labors are usually very rapid ones, the child meeting its death then from blows, falls, or other injury. As a general rule, we may say that *vagitus uterinus* can be supposed to have occurred only—1, When the labor has been very tedious; 2, When the liquor amnii has been long discharged; 3, When there are no objective signs of violence having been employed upon the child; 4, When none of the signs of the accidents are present which commonly result from a tedious labor, especially apoplexy. Dr. Landsberg, however, quotes a case related by Zitterland (Henke's Zeitschrift, 1823, p. 237), in which the child is stated to have cried long and distinctly before the least signs of labor came on, the first pains, and the opening of the os uteri not taking place until the following day. The child was still-born, but restored to life.

Two other cases, besides the one quoted in our last, have been recently published. One of these occurred to Dr. Falkenbach, who, during the operation for turning for a cross-birth, and while the child was undoubtedly entirely within the uterus, heard it cry loudly several times, as did other persons in the room. The other case, too, related by Dr. Koehler, occurred during the attempt to bring down a second foot in turning. The tone of voice was just like that peculiar one uttered by the new-born child, but somewhat duller, as though the child were in a cellar or lower apartment. It continued crying at intervals for two or three minutes. After this, the delivery was accomplished quickly, until the shoulders arrived in the pelvis, which was rather narrow, while the child was a large one. Both they and the head passed through with great difficulty, and the child was born dead, beyond recovery. The child's cries, while *in utero*, were heard by three other people in the room, as well as by the midwife, who was hard of hearing. The chest was examined twenty-four hours afterwards, and was found to be well expanded. The convexity of the diaphragm upwards was less than in the fœtal state. Although the lungs did not completely fill the cavity of the chest, they did so to a considerable extent, in part covering the pericardium. The lungs, heart, and a very large thymus gland were removed, and, being placed unseparated in a small vessel of cold water, swam completely. The lungs were of a bright red, with here and there some blueish spots. On incision they crepitated, and some foam and a little blood flowed out. When cut under water, large air-bubbles rose to the surface, and when divided into small portions, each of these swam. The air-cells of the periphery had alone not become completely filled with air, whence the blueish spots found there; but still, not the smallest portion of the structure sank.—*Henke's Zeitschrift, Suppl.*

[This last case is certainly one of high interest in reference to the inferences to be drawn from the application of the hydrostatic test. A train of circumstances can be easily imagined, which might have led to an unjust charge of infanticide, if this test possessed the judicial authority which it once did.—*Ibid.*

